# Sex Dependence in Control of Renal Haemodynamics and Excretion in Streptozotocin Diabetic Rats—Role of Adenosine System and Nitric Oxide

**DOI:** 10.3390/ijms25147699

**Published:** 2024-07-13

**Authors:** Marta Kuczeriszka, Leszek Dobrowolski

**Affiliations:** Department of Renal and Body Fluid Physiology, Mossakowski Medical Research Institute, Polish Academy of Sciences, A. Pawinskiego 5, 02-106 Warsaw, Poland; mkuczeriszka@imdik.pan.pl

**Keywords:** nitric oxide, female, diabetes, sex difference, oxidative stress, renal microcirculation, tubular transport

## Abstract

Recently, we compared an interplay of the adenosine system and nitric oxide (*NO*) in the regulation of renal function between male normoglycaemic (NG) and streptozotocin-induced diabetic rats (DM). Considering the between-sex functional differences, e.g., in the *NO* status, we present similar studies performed in female rats. We examined if the theophylline effects (non-selective adenosine antagonist) in NG and DM females with or without active *NO* synthases differed from the earlier findings. In anaesthetised female Sprague Dawley rats, both NG and DM, untreated or after *NO* synthesis blockade with L-NAME, theophylline effects, on blood pressure, renal hemodynamics and excretion, and renal tissue *NO* were investigated. Renal artery blood flow (Transonic probe), cortical, outer-, and inner-medullary flows (laser-Doppler technique), and renal tissue *NO* signal (selective electrode) were measured. In contrast to males, in female NG and DM rats, theophylline induced renal vasodilation. In *NO*-deficient females, theophylline induced comparable renal vasodilatation, confirming the vasoconstrictor influence of the renal adenosine. In NG and DM females with intact *NO* synthesis, adenosine inhibition diminished kidney tissue *NO*, contrasting with an increase reported in males. Lowered baseline renal excretion in DM females suggested stimulation of renal tubular reabsorption due to the prevalence of antinatriuretic over natriuretic tubular action of adenosine receptors. An opposite inter-receptor balance pattern emerged previously from male studies. The study exposed between-sex functional differences in the interrelation of adenosine and *NO* in rats with normoglycaemia and streptozotocin diabetes. The findings also suggest that in diabetes mellitus, the abundance of individual receptor types can distinctly differ between females and males.

## 1. Introduction

Diabetes is a chronic metabolic disease characterized by elevated blood glucose levels (hyperglycaemia), which contributes to the development of multi-organ complications that pose a serious threat to patients’ lives and generate huge social and economic costs. Diabetes complications include retinopathy and nephropathy, heart disease, stroke, and diabetic foot syndrome. In addition to hyperglycaemia, a high level of oxidative stress is described in diabetes. Simultaneously, in diabetic patients and in animal experimental diabetic models, the level of nitric oxide (*NO)* and its bioavailability was found to be lower than in normoglycaemia. *NO* is an established important factor in the control of vascular tone [1,2], particularly in the kidney. Inhibition of *NO* synthesis results in an increase in renal vascular resistance (RVR), a decrease in renal plasma flow (RPF) and sodium excretion (U_Na_V), and, as a consequence, in hypertension [3].

The vascular endothelium is the main site of *NO* production. The actual activity of endothelial *NO* synthases (NOSs) depends on the bioavailability of several cofactors, e.g., tetrahydrobiopterine (BH_4_) [4]. Interestingly, sex-specific differences were described in *NO* bioavailability and NOS protein expression, observed both in Sprague Dawley and spontaneously hypertensive rats [3,5,6].

The changes in *NO* synthesis and bioavailability, in combination with hyperglycaemia, are responsible for endothelial damage and dysfunction leading to an increase in vascular resistance, hypertension, and proteinuria. All these processes contribute to or are an effect of oxidative stress, which per se is also responsible for the mentioned negative phenomena.

In the kidney, reactive oxygen species (ROS) interact with *NO*. Superoxide (O_2_^−^) and its derivative molecules, such as hydrogen peroxide (H_2_O_2_) and peroxynitrite (ONO_2_^−^) are known to regulate solute and water reabsorption and thereby help maintain electrolyte homeostasis and extracellular fluid volume [7]. Also, *NO* has a strong natriuretic and diuretic action, as a result of its inhibitory effect on proximal tubular water and Na^+^ transport [7,8]. ROS were shown to constrict the systemic and intrarenal arterial vasculature by modulation of the action of other endothelium-derived factors. On the other hand, optimal ROS production is required for normal cell signalling as they serve as a second messenger for activation of some pathways involved in cell growth, inflammation, apoptosis, and cell differentiation.

Adenosine (Ado) dilates the vessels in most tissues, while in the kidney, it can cause both vasoconstriction and vasodilatation, depending on the prevailing stimulation of adenosine A1 or A2 receptors (A1R, A2A/BR), respectively [9]. The renal effect of another Ado receptor, A3, has been less investigated. The mediator of the Ado vasodilatory effect could be *NO* as was shown previously in the study on the action of endogenous or exogenous adenosine [10]. The *NO*–adenosine relationship may be important, especially in the medulla, which operates under relative hypoxia and is susceptible to ischaemic damage [11]. It was demonstrated that A2AR-induced efferent arteriole vasodilation through *NO* could counteract the vasoconstriction mediated by the adenosine A1 receptor in the afferent arterioles. This mechanism is thought to maintain the glomerular filtration rate within the normal range, but it is abolished in diabetes [12]. Moreover, some data indicate that A2AR activation promotes the increase in ROS generation, but may also activate eNOS, causing a *NO* production increase, which may impair the deleterious effects mediated by ROS and oxidative stress [13].

The abundance of particular types of Ado receptors (which belong to the P1 receptor subtypes family, P1R) differs between the renal cortex and medulla. A higher A1R level in the cortex compared to the medulla and an opposite pattern for A2R expression are usually reported [14]. It is also suggested that the abundance of both receptor types is distinctly altered in pathological states, e.g., in diabetes mellitus (DM), as reviewed by Burnstock and Novak (2013) and Antonioli et al. (2015) [15,16]. However, the limited data regarding P1R in diabetes come from a study performed in male animals. Also, tubular transport is under contrary control of P1R: the net effect of A2R activation is natriuresis, whereas A1R activation is antinatriuretic.

P1R also participates in the regulation of glucose metabolism and therefore has a role in diabetes mellitus. For instance, Ado causes a rapid increase in carrier-mediated glucose uptake and acts via receptors linked to a signalling pathway that involves intracellular cyclic adenosine monophosphate (cAMP) production [17].

While it was shown that Ado through its P1R can contribute to the maintenance of glucose homeostasis and modulate diabetes, the role of individual P1R subtypes is still unclear [17]. Most of the relevant experiments were performed on male animals. Single studies on purinergic receptor activity in females were performed in vitro. For instance, in women with gestational diabetes, hyperglycaemia was associated with elevated gene expression levels of the A2BRs in leukocytes [17].

Recently, we showed a close interrelationship between *NO* and Ado receptors both in NG and DM rats (adenosine-caused vasodilation seemed to be *NO*-dependent); however, the study was performed in male rats only [18]. It will be noticed here that sex-dependence exists in NOS activity, which is higher in females than in males, and the result might be greater *NO* synthesis in the former, possibly triggering excessive synthesis of ROS. There is a growing awareness of the need to perform experiments on animals of both sexes. The American Physiological Society and the British Pharmacological Society recently recommended that sex should no longer be ignored as an experimental variable to be tested. Indeed, this is crucially important in preclinical research as a prerequisite for the successful translation of the results into clinical practice [19,20].

Considering such convincing recommendations, we have decided to extend our earlier pertinent research performed in male rats and supplement them with studies with their female counterparts, both normoglycaemic and with experimentally streptozotocin-induced diabetes (STZ-induced DM). The focus was to examine if the effect of theophylline (Theo, a non-selective Ado antagonist) in NG and DM females with or without NOS chronic blockade differed from the earlier findings in the males. Theo, a nonselective Ado receptor antagonist with very low affinity to A3R compared to the A1R and A2R subtypes of P1R [21], was often shown to inhibit adenosine-induced vascular actions in the kidneys of dogs and rats [22]. The diuretic and natriuretic effects of theophylline are well-known and inhibition of Ado receptors is critical for its natriuretic action [23].

## 2. Results

### 2.1. Chronic Study

During the chronic part of the project, several parameters were tested. For convenience and clarity of presentation, we will show only those in which significant changes or differences were observed.

Table 1 summarises the data from chronic observations (2 weeks = 14 days) of NG and DM rats (i.e., after buffer or STZ injection), with or without L-NAME treatment for the four days before the acute experiment (+/−L-NAME_4_).

#### 2.1.1. Phase of the Oestrus Cycle, Body Weight (Bwt), Blood Glucose Level (BG)

In order to determine the phase of the oestrous cycle, a vaginal smear was performed: all females were found to be in the proestrus phase. This could be ensured due to good animal synchronization: the rats shared the same cage prior to the start of the experiment.

In NG females, a gradual increase in Bwt was observed over 14 days of observation. In DM, Bwt was not different between day 0 and 14 (237 ± 3 vs. 233 ± 8 g), except for a transient decrease noted on day 7 (227 ± 4 g, *p* < 0.05 vs. day 0). Noteworthy chronic NOS inhibition (L-NAME_4_) did not alter Bwt gain in NG and DM rats.

BG remained stable in NG rats throughout two weeks. As expected, in DM rats, glycaemia increased beginning from the third day after STZ injection (430 ± 16 mg/dL, *p* < 0.05 vs. day 0) and was maintained greater until the end of the observation. L-NAME_4_ treatment did not affect glycaemia in NG or DM rats; however, a visible higher BG (by 30 mg/dL compared to day 10) was shown in DM group.

#### 2.1.2. Blood Parameters

During chronic observation prior to L-NAME treatment, haematocrit (Hct) values increased in NG but did not alter in DM animals. However, after L-NAME_4_ treatment, Hct was not changed in NG, but in DM females it was slightly higher vs. day 0 (*p* < 0.02). 

There was no significant difference in plasma osmolality (P_osm_) between NG and DM rats without L-NAME treatment; in DM females, a 5% increase was seen on day 10 (*p* < 0.01). Notably, in both groups, L-NAME_4_ caused a significant increase in P_osm_ vs. day 10th (by 7%, *p* < 0.03).

In NG animals, plasma sodium concentration (P_Na_) did not alter along the chronic observation, both without and with L-NAME_4_. However, in DM animals, P_Na_ was significantly lower before L-NAME treatment in comparison with the respective day 0 (*p* < 0.03), and also lower than P_Na_ in NG animals (*p* < 0.04). Plasma potassium concentration (P_K_) both in NG and DM animals did not alter for the whole time of chronic observation (14 days), similarly without and with L-NAME_4_.

#### 2.1.3. Daily Water Intake and Urine Excretion

As expected, in NG animals prior to L-NAME treatment, no changes in water intake were observed, whereas in DM animals, about a 3-fold increase (*p* < 0.001) was seen. L-NAME_4_ caused an increase (vs. day 10) in the volume of water drunk (not significant).

In NG animals, daily urine flow did not alter during chronic observation, irrespective of L-NAME treatment. In DM animals about an 8-fold increase (*p* < 0.0001) of diuresis was seen (day 10), and chronic addition of L-NAME caused a 25% increase in diuresis (not significant in comparison with the respective day 10).

Daily total solute excretion (U_osm_V) during chronic observation before L-NAME treatment (day 10) decreased in NG (*p* < 0.001), whereas in DM animals, it increased about 6-fold (*p* < 0.001) compared with day 0. U_osm_V significantly differed between groups (*p* < 0.001). Chronic addition of L-NAME restored solute excretion towards the basal value in NG but did not alter it significantly in the DM group. However, the U_osm_V difference between NG and DM females proved significant.

The pattern of daily U_Na_V changes was similar to that of U_osm_V. During chronic observation before L-NAME treatment, U_Na_V decreased in NG (*p* < 0.001), whereas in DM animals, it increased about twice (*p* < 0.002) compared with day 0. The U_Na_V differed significantly between groups (*p* < 0.001). Chronic addition of L-NAME restored sodium excretion towards the basal value in NG but did not alter it in the DM group. However, the U_Na_V difference between NG and DM females proved significant.

### 2.2. Acute Experiments

#### 2.2.1. Effects of L-NAME_4_ Pretreatment in NG and DM Rats on Baseline Haemodynamics and Renal Circulation

The data on basal haemodynamics, renal perfusion, and excretion are collected in Table 2. No difference in MABP between NG and DM females without L-NAME_4_ treatment was observed during control periods. However, as expected, both in NG and DM groups without pretreatment, MABP was lower compared with those receiving L-NAME_4_: in NG by 20% (123 ± 3 vs. 149 ± 2 mmHg, *p* < 0.001), and in DM by 15% (124 ± 2 vs. 143 ± 4 mmHg, *p* < 0.0001).

Basal RBF and CBF were significantly higher in NG than in DM females (4.7 ± 0.3 vs. 3.8 ± 0.2 mL/min/g, *p* < 0.01 and 655 ± 25 vs. 535 ± 15 PU, *p* < 0.0002, respectively). As expected, L-NAME_4_ pretreatment in NG and DM groups lowered baseline RBF (by 42% and 40%, respectively, *p* < 0.0001), and CBF (by 30% and 21%, respectively, *p* < 0.0001). 

Interestingly, both basal OMBF and IMBF were unaffected by diabetes or L-NAME_4_ pretreatment. 

#### 2.2.2. Effects of L-NAME_4_ Pretreatment in NG and DM Rats on Baseline Renal Excretion

Baseline diuresis differed between NG and DM females (10.8 ± 1.4 vs. 7.0 ± 0.8 µL/min/g, *p* < 0.03). L-NAME_4_ treatment lowered urine flow in NG females (7.3 ± 0.8 µL/min/g, *p* < 0.05) but did not alter it in DM animals (8.5 ± 3.2 µL/min/g, NS). This was so despite the post-L-NAME_4_ blood pressure elevation (no pressure diuresis). 

There was also a baseline U_osm_ difference between NG and DM females (710 ± 35 vs. 1000 ± 35 mosmol/kg H_2_O, respectively, *p* < 0.0001). Again, L-NAME_4_ treatment lowered urine osmolality in NG females (580 ± 25 mosmol/kg H_2_O, *p* < 0.002) but did not alter it in DM animals (1045 ± 170 mosmol/kg H_2_O, NS, U_osm_ data shown in Supplementary Files). However, baseline U_osm_V did not differ between the NG and DM groups. In L-NAME_4_-treated NG females, U_osm_V was lower (6.8 ± 0.7 vs. 4.1 ± 0.4 µosmol/min, respectively, *p* < 0.002), whereas in DM rats, it was not altered. 

Interestingly, U_Na_V was significantly higher in NG than in DM females (1.4 ± 0.4 vs. 0.5 ± 0.1 µmol/min/g, respectively, *p* < 0.02). In L-NAME_4_-treated NG females, U_Na_V was 50% lower (0.7 ± 0.1 µmol/min/g, *p* < 0.05), whereas in DM, it was 3-fold higher than in their non-treated counterparts (1.7 ± 0.3 µmol/min/g, *p* < 0.001). 

Also, U_K_V was significantly higher in NG than in DM females (0.9 ± 0.1 vs. 0.3 ± 0.1 µmol/min/g, respectively, *p* < 0.0001). In L-NAME_4_-treated NG females, U_K_V was lower by 40% (0.6 ± 0.1 µmol/min/g, *p* < 0.03), whereas in DM, it was twice as high as in their non-treated counterparts (0.6 ± 0.1 µmol/min/g, *p* < 0.002). 

#### 2.2.3. Effects of Theophylline on MABP, Heart Rate, and on Renal Total and Regional Perfusion in NG and DM Rats Untreated or Pre-Treated with L-NAME_4_

**Figure 1:**
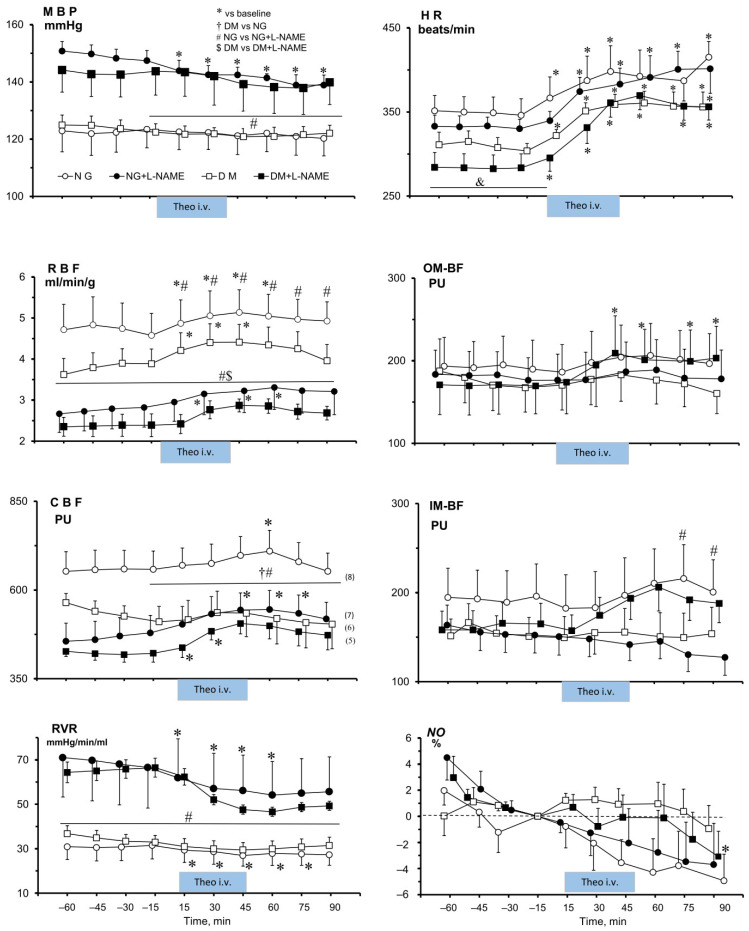
Effects of theophylline (Theo) on MABP, HR, renal haemodynamics, and the in situ tissue *NO* signal in the renal medulla of normoglycaemic (NG) and hyperglycaemic female rats (DM), untreated or pre-treated with L-NAME. Means ± SEM. MABP—mean arterial blood pressure; HR—heart rate; RBF—whole-kidney blood flow; CBF, OM-BF, and IM-BF—cortical, outer- and inner-medullary blood flow, respectively; RVR—renal vascular resistance. *—significantly different from the respective baseline; †—significantly different from the NG rats; #—significant difference between NG-non-treated and L-NAME-pre-treated rats; $—significant difference between DM-non-treated and L-NAME-pre-treated rats.

Figure 1 shows that Theo affected MABP in NG-L-NAME_4_ rats only, with a gradual decrease from 147 ± 4 to 139 ± 4 mmHg. Interestingly, HR was increased by Theo both in NG and DM females (by 16–26%), irrespective of L-NAME_4_ treatment; significant elevation above baseline persisted after cessation of drug infusion.

After Theo infusion, RBF increased transiently, both in NG and DM rats (by 17 ± 4% and 23 ± 8%, respectively). However, after L-NAME_4_ pretreatment, Theo increased RBF (by 18 ± 4%) only in NG rats (*p* < 0.02), whereas in DM rats, the increase was smaller and not significant (21 ± 12%, *p* = 0.09) (Figure 1). Similarly, Theo increased CBF in untreated and L-NAME_4_-pre-treated NG animals; however, the changes were smaller than those for RBF (11 ± 2% and 18 ± 4%, *p* < 0.0003 and 0.01, respectively). On the other hand, in DM rats, Theo did alter CBF (by 16 ± 4% (*p* < 0.03) only in the L-NAME_4_ group.

The changes in medullary blood perfusion differed from those seen in the cortex. Medullary blood flow was affected by Theo only in DM rats treated with L-NAME_4_; however, only the increase in OM-BF (by 23 ± 5%) was significant (*p* < 0.01). In NG females, only the Theo effects on IM-BF differed between the untreated and L-NAME-treated groups.

Since in NG, NG+L-NAME_4_, and DM+L-NAME_4_, the RBF increase induced by Theo occurred with no change or a decrease in MABP, the calculated renal vascular resistance (RVR) was decreased in NG rats only and persisted below the baseline value even after cessation of the drug infusion. Dissimilarly, in DM rats, only in those pre-treated with L-NAME_4_ did Theo evoke an RVR decrease by 28 ± 5% (*p* < 0.01), without recovery to the baseline value till the end of the experiment (Figure 1).

#### 2.2.4. Effects of Theophylline (Theo) on Renal Tissue *NO* Signal in NG and DM Female Rats, Untreated or Pre-Treated with L-NAME_4_

Theo infusion significantly decreased the renal medullary tissue *NO* signal in NG rats, by 5 ± 2%, *p* < 0.05. This effect was not clearly altered by L-NAME pretreatment; however, the Theo-induced change by 4 ± 3% was not significant (Figure 1).

Moreover, we found here that an i.v. bolus of L-NAME, given after completing experiments with control (no Theo treatment) NG and DM rats chronically pre-treated with different doses of L-NAME, induced only a minimal 1–2% decrease in the tissue *NO* signal, quite similar to that in the NG and DM groups. This indicates that the chronic dosage, higher in diabetic than in normoglycaemic animals, was adequate and maximally efficient in both groups. We have not attempted to make any quantitative inter-group comparisons of basal or post-treatment *NO* signal values: while the method enables reliable estimation of changes in bioavailable tissue *NO* during a given experiment, it may be of doubtful value as a measure of absolute *NO* concentration

#### 2.2.5. Effects of Theophylline on Renal Excretion in NG and DM Female Rats, Untreated or Pre-Treated with L-NAME

**Figure 2:**
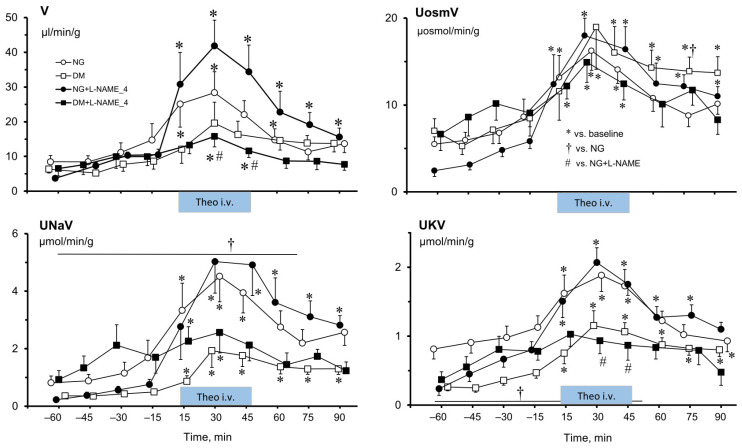
Effects of theophylline (Theo) on renal excretion in normoglycaemic (NG) and hyperglycaemic female rats (DM), untreated or pre-treated with L-NAME. Means ± SEM. V—urine flow; U_osm_V, U_Na_V, U_K_V—total solute, sodium and potassium excretion, respectively. *—significantly different from the respective baseline; †—significantly different from the NG; #—significant difference between NG and DM pre-treated with L-NAME.

Similarly to the effects of Theo on renal haemodynamics (Figure 1), the changes induced in renal excretion parameters were unidirectional (Figure 2). Usually, an increase in V, U_osm_V, U_Na_V, and U_K_V during Theo infusion was seen in NG and DM rats, irrespective of L-NAME_4_ pretreatment. The exception was U_osm_, which in NG rats was elevated after Theo infusion, and similar to that in untreated and pre-treated rats. U_osm_ did not change in DM animals (data shown in Supplementary Files).

Urine flow (V) was increased by Theo in NG and even more so in the NG+L-NAME_4_ group (by 156 ± 33% vs. 263 ± 38%, respectively, *p* < 0.05). On the other hand, in DM rats, Theo-induced V elevation was smaller in the L-NAME_4_-treated group (177 ± 63% vs. 83 ± 19%, respectively, *p* < 0.002).

As seen also with V, Theo increased U_Na_V in the NG and NG+L-NAME_4_ groups (by 330 ± 70% vs. 790 ± 185%, respectively, *p* < 0.04), and in the DM and DM+L-NAME_4_ groups (by 385 ± 125 vs. 75 ± 15%, respectively, *p* < 0.05).

The increase in potassium excretion (U_K_V) caused by Theo was also reduced by L-NAME_4_ treatment, in NG from 220 ± 30% to 135 ± 40%, respectively (change not significant), whereas the difference between DM and DM+L-NAME_4_, by 190 ± 50 vs. 55 ± 20%, respectively, was significant (*p* < 0.03). A similar tendency of post-Theo changes was seen for U_osm_V, both in NG+L-NAME_4_ and in DM and DM+L-NAME_4_ groups. However, these differences were not significant.

## 3. Discussion

The study aimed to compare the role of the adenosine system in its interrelation with *NO* in the control of renal and systemic circulation and renal excretion between normoglycaemic and streptozotocin diabetic female rats. In most of relevant studies, males were used, to avoid the effect of the oestrous phase on the results obtained. In the current study, females were kept in synchronised groups of animals, which enabled synchronisation of the oestrus cycle and ensured a comparable hormonal environment in all groups.

Discussed below are the data from female rats and how they compare with the results of our recent studies performed in males [18,24].

### 3.1. Effects of STZ-Induced Hyperglycaemia on Metabolic and Renal Excretion Parameters

An increase in food intake without any weight increase was shown in hyperglycaemic rats independent of sex, possibly depending on the STZ-induced hypoinsulinaemia. In addition to the loss of muscle mass, the relative Bwt lowering may have been due to dehydration caused by osmotic diuresis. Possible dehydration was probable because of the plasma osmolality increase in DM; in NG animals, no such alteration was seen. The same pattern of changes was observed in females and, as reported earlier [18,24], in males. However, there were no glycaemia-dependent changes in P_Na_ and P_K_ in female rats, whereas in males, a decrease in P_K_ was shown at the end of chronic observation.

### 3.2. Effects of STZ-Induced Hyperglycaemia on Systemic and Renal Haemodynamics and Renal Excretion

In the females, baseline MABP did not differ between NG and DM groups, as was also seen in males. As expected, L-NAME only tended to increase baseline MABP, both in NG and DM rats. Dissimilarly, in NG and DM males, the difference between the untreated and L-NAME groups was significant. Thus, under baseline conditions (without Ado blockade), the tonic influence of *NO* could be more important in female than in male rats.

As in males, in females, baseline HR was slightly higher in NG than in the DM group. However, after L-NAME, in contrast to males, HR was lower than in untreated NG and DM females.

As in males, baseline RBF was in females slightly higher in the NG than in the DM group. As expected, after L-NAME, as in males, baseline RBF was in both NG and DM female rats significantly lower, most probably due to the lack of vasodilatory *NO* after chronic NOS inhibition.

In females, baseline RVR did not differ between NG and DM rats and, as expected, significant increases were observed after L-NAME in both groups. A similar pattern of changes was shown previously in our study with male rats. Nevertheless, a higher RVR might be expected in females than males, as was pointed out in some reviews [25,26].

Taken together, data from our studies indicate that any differences in the baseline (prior to the acute experiment) vascular responses between female and male rats were negligible, which suggests that the vasodilator influence of *NO* was similar in either sex.

#### 3.2.1. Renal Regional Blood Perfusion

Interestingly, in the females, neither basal OMBF nor IMBF were affected by diabetes or L-NAME treatment, despite evidence that the renal medulla is characterized by a high NOS expression and activity, especially nNOS [27,28]. We reported that in normal male rats, *NO* bioavailability is much higher in the renal medulla compared to the cortex [29]. It is worth mentioning that in Wistar NG males, blockade of NOS activity tended to decrease medullary perfusion, in parallel with peripheral vasoconstriction (increase in MABP) and a decrease in RBF [30].

Regarding the current findings, one can speculate that the applied dose of L-NAME_4_ was too low to effectively block the local medullary NOS activity. However, the same dose evidently caused peripheral vasoconstriction (increased MABP); simultaneously, it reduced RBF (i.e., perfusion of the whole renal cortex) and CBF (perfusion of the superficial cortex). In general, irrespective of sex, NOS activity in the medulla is higher than in the kidney cortex, and in females, it could be additionally elevated by short-term diabetes [5,6]. It is unclear why in SD rats of both sexes, chronic L-NAME treatment caused peripheral and renal cortical vasoconstriction, whereas it did not modify the medullary perfusion. It seems that irrespective of sex, renal medullary circulation is well preserved. This could be connected with its postulated role in the long-term control of blood pressure [31].

#### 3.2.2. Renal Excretion

Unexpectedly, in female rats, basal urine flow was slightly higher in the NG than in the DM groups, in contrast to what was seen in respective male groups. In the latter, distinctly higher (at least twice) baseline diuresis was shown in DM, as compared to NG [18,24]. Noteworthy, data from chronic observations of both sexes showed a several times higher renal excretion in DM compared with the NG group. However, such dissimilarity between groups was preserved after anaesthesia in males only [18,24] and evidently not in females. Astonishingly, chronic NOS inhibition did not affect urine flow in female rats, even though L-NAME treatment was associated with a blood pressure increase (~25 mmHg), and a pressure-dependent increase in diuresis could be expected. The same puzzling response was also noted in the study with male rats [18].

Surprisingly, basal renal sodium excretion was higher in NG than in DM females, in contrast to the distinct opposite difference in male rats. Moreover, in females, chronic blockade of NOS activity reversed the sodium excretion response pattern, whereas in male rats, there was no difference in sodium excretion between NG and DM rats [18]. Notably, this occurred while the baseline blood pressure was increased, both in females and males, independent of glycaemia level. Noteworthily, in experiments with acute intravenous L-NAME delivery to male rats, a significant increase in sodium excretion without changes in blood pressure was shown. This accords with the notion of the specific effect of L-NAME on tubular transport [32]. The differences between female and male renal excretion in NG and DM rats, as described above, are schematically summarized in Table 3.

Taken together, these findings indicate that in females, diabetes affects renal excretion differently than in males, especially in the case of sodium and potassium excretion and urine osmolality. Apparently, long-lasting hyperglycaemia causes in females an increase in reabsorption of sodium and potassium, probably depending on the tubular action of *NO.*

### 3.3. Effects of Ado Receptor Blockade in NO-Intact Rats on Systemic and Renal Haemodynamics, and on Renal Excretion and Tissue NO

As also seen in males, nonspecific A1 and A2 receptor inhibition with Theo did not modify MABP in NG and DM females. Thus, irrespective of sex, under our experimental conditions (anaesthesia and surgery), there was no apparent basal tonic influence of the Ado system on total peripheral vascular resistance (TPVR).

#### 3.3.1. Renal Haemodynamics

Theo administration induced an increase in whole-kidney perfusion (RBF), both in NG and DM female rats. This was in striking contrast to the opposite change between NG and DM males, an increase vs. decrease, respectively [18,24]. Consequently, in females, the post-Ado receptor blockade changes in RVR did not differ between the NG and DM groups. Again, this was in contrast to what was seen in males but, as could be expected, a decrease in RVR in NG vs. an increase in DM was shown [18,24].

Interestingly, the described between-sex variation in the renal haemodynamics after Ado receptor blockade was not seen within the medulla: OMBF remained unchanged in females, whereas it tended to decrease in males, irrespective of the glycaemia. Dissimilarly, IMBF tended to increase. These findings support the view that the Ado system contributes to the control of blood perfusion, but differently in the cortex and medulla. [18,24,33,34]. However, in diabetes, the dissimilarity between the cortical and medullary circulation appears to be modulated by sex.

#### 3.3.2. Renal Excretion

Acute intravenous delivery of a non-selective purinergic receptor antagonist (Theo) increased renal excretion (V, U_osm_V, U_Na_V) both in NG and DM female rats, similar to what was observed in male rats [18]. Notably, in NG female rats, the natriuresis increase was greater than in DM, whereas no such difference was shown between NG and DM males. Thus, in the situation without *NO* blockade, purinergic receptors contribute to the control of tubular transport in both sexes independent of glycaemia. However, differently than in males, Ado blockade causes a greater reduction in sodium reabsorption and, consequently, a higher U_Na_V in NG than in DM females. 

#### 3.3.3. Tissue *NO*

Surprisingly, contrary to the increase in the renal tissue *NO* signal observed in males [18], acute non-selective antagonism of purinergic receptors did not induce any such change in NG and DM female rats. In the latter, rather a tendency to a decrease in DM and a slight decrease in tissue *NO* was noted (Figure 1). This effect could be expected because inhibition of A2R by Theo could cause a decrease rather than an increase in kidney tissue availability of *NO*.

### 3.4. Effects of Ado Receptor Blockade in NO-Deficient Rats on Systemic and Renal Haemodynamics, and on Renal Excretion and Tissue NO

#### 3.4.1. Unexpected Post-Theo Decrease in MABP in NG Female Rats

Under conditions of impaired *NO* synthesis in NG females, Theo caused a significant and sustained decrease in MABP, whereas in the DM group, blood pressure remained unchanged. This suggests an important *NO* participation in MABP control in NG females. This contrasts with the unresponsiveness of MABP in male NG rats, both without and with *NO* inhibition [18].

Why in the *NO*-deficient normoglycaemic rats was the overall TPVR sensitive to the blockade of Ado receptors? Any association of such sensitivity with A2 blockade seems unlikely: the role of the A2 effect (*NO* stimulation) was excluded by *NO* blockade before Theo administration. Nevertheless, the influence of *NO*-mediated A2 receptors must still be considered: the reduction in MABP may have been due to a reduction in cardiac output. Adenosine can enhance coronary circulation in rats by stimulating A2 receptor activity, also in the absence of *NO*. It was reported that A2 receptors (both A and B subtypes) can evoke *NO*-independent smooth muscle relaxation in coronary arteries [35]. Therefore, an inhibition of these receptors with Theo may induce coronary vasoconstriction leading to a decrease in cardiac output and followed by a fall in blood pressure. However, the increase in heart rate shown in all our female groups, irrespective of glycaemia, does not support this hypothesis. Alternatively, TPVR decrease and blood pressure drop could be explained by elimination by Theo of the vasoconstrictor effect of A1R. However, MABP did not decrease in NG rats without *NO* blockade. Perhaps if the decrease was the result of a simultaneous inhibition of A2R-induced vasodilation, then a zero net effect would be likely. The same explanation would also be valid for DM rats.

The unusual hypotensive response to Theo seen in NG rats with *NO* deficiency was not due to a different basal TPVR because the baseline post-L-NAME level of BP was almost the same in NG and DM rats, and the RVR was comparable (Table 2). One can speculate that the reason could be the higher basal density of A1R in NG compared to DM rats.

In general, our results do not accord with the evidence of Ado-induced relaxation (rather than contraction) reported in a recent study of endothelium-denuded rat aorta isolated from nondiabetic males [36]; however, caution is needed in attempts to compare the results of ex vivo and whole-animal studies.

In addition to the unusual post-Theo blood pressure decrease, the *NO*-deficient female NG rats showed a similarly unusual decrease in renal inner-medullary perfusion (IMBF). Interestingly, such a pattern of post-Theo changes in MABP and IMBF was reported in males but only in those with hyperglycaemia. We suggest that it was secondary to a decrease in MABP because of impaired autoregulation of IMBF. Indeed, such impairment in the face of blood pressure alterations was repeatedly reported [31,37].

#### 3.4.2. Renal Blood Perfusion

Interestingly, in the females, contrary to the tubular effects of Theo (see below), the hemodynamic effects were not dependent on NOS activity, unlike in the study with male rats [18]. In addition, we saw that under conditions of systemic *NO* deficiency, Ado receptor blockade induced renal vasodilatation and increased perfusion, probably due to abolishment of baseline (pre-Theo) vasoconstriction. The effect tended to be more pronounced in NG rats. Remarkably, the pronounced decrease in RVR was similar here to the situation without *NO* blockade, when Ado receptor blockade slightly decreased RVR. On the whole, under conditions of *NO*-deficiency, there was only a slight difference between NG and DM rats in the response of the renal circulation to Ado receptor blockade, which speaks for a comparable vasoconstrictor influence of the Ado system in females, independent of glycaemia (Figure 1). Our results from Ado inhibition experiments do not quite accord with those obtained with the application of exogenous Ado. In normoglycaemic males, the renal vasculature was found more sensitive to adenosine-mediated vasoconstriction when *NO* synthases were inhibited [38], whereas in females, we did not show such dependency. Similarly, the kidneys of streptozotocin-diabetic rats were clearly more responsive to the vasoconstrictor action of Ado in *NO*-deficient animals [10], which was not observed in our study with females.

Noteworthily, in diabetic females under conditions of systemic *NO* deficiency, a slight increase and a tendency to an increase after Ado receptor blockade were seen for OMBF and IMBF, respectively. This suggests that hyperglycaemia can evoke some changes in Ado receptor contribution to control medullary circulation, which was not seen in males [18]. The post-Theo increase in perfusion seems to be not simply the effect of elimination of A1R because it also occurred in the NG and DM females given Theo, in which no increase in OMBF or IMBF was seen.

#### 3.4.3. Renal Excretion

In females, the Theo effects after NOS inhibition differed between NG (an increase) and DM (a decrease), whereas no such difference was earlier reported in males. Similar post-Theo effects were shown in females for sodium and potassium but not for total solute excretion, which showed no difference between NG and DM, both with and without *NO* blockade. This suggests that *NO* influence on the tubular action of purinergic receptors in female rats is modified by glycaemia.

#### 3.4.4. Tissue *NO*

Remarkably, under conditions of inhibited *NO* synthesis, acute non-selective antagonism of purinergic receptors did not induce any changes, similarly in NG and DM female rats. It was, in some sense, unexpected in the NG group because a parallel decrease in MABP and impairment of IMBF (possible reduction in tissue anoxia) might be a stimulus for *NO* release from a source other than NOS, e.g., from S-nitrosothiols, a main form of *NO* storage within the vasculature [39]. In males, the same direction of changes in blood pressure and medullary perfusion (actually more pronounced) was associated with an increase in tissue *NO* in the DM group. On the other hand, in the NG group, Theo did not alter MABP and IMBF, nor tissue *NO* [18].

## 4. Materials and Methods

### 4.1. Animals

All protocols were approved by the Second Local Ethical Committee, Warsaw, Poland (number 156/2021), and were in accordance with the National Institutes of Health Guide for the Care and Use of Laboratory Animals and the European Union Directive 63/2010. Female Sprague Dawley (Tac:Cmd:SD) rats, diabetic (DM) or non-diabetic (NG) were used in this study. Hyperglycaemia was induced in rats aged 6–7 weeks obtained from the intramural animal breeding house. The animals were housed in groups of 2–4, under 12:12 h light/dark cycle, and had free access to tap water and standard rat chow (dry pellets with 0.25% Na *w*/*w*, SSINFF GmbH, Soest, Germany). Female rats were kept in stable groups, both during breeding and before the primary chronic experiment, which ensured that the oestrus cycle was synchronized. Additionally, at the beginning of the experiment, a vaginal smear was performed to determine the phase of the cycle.

### 4.2. Chronic Studies

#### 4.2.1. Induction of Diabetes

Diabetes was induced with streptozotocin (STZ i.p.; 60 mg/kg, Santa Cruz Biotechnology, Inc., Dallas, TX, USA), dissolved in citrate buffer (0.05 mol/L, pH 4.5) directly before injection. The rats’ body weight (Bwt) and blood glycaemia (BG) were determined beginning from the day before STZ injection until the acute experiment. On days 3, 7, and 10 or 14 after STZ administration, glucose level was measured, following 2 h food deprivation. The animals were considered diabetic if blood glucose was higher than 300 mg/dL measured 72 h after STZ injection and remained so elevated until the end of the observation. In order to define the phase of the oestrus cycle, a vaginal smear was taken in the chronic part of the experiment.

#### 4.2.2. L-NAME Pretreatment

Prior to the acute experiment, in randomly selected NG and DM animals, N(G)-nitro-L-arginine methyl ester (L-NAME; Sigma, Poznań, Poland), a nonselective NOS inhibitor, was administered orally for four days (L-NAME_4_) by admixing the powdered substance, 5 mg/100 mL, to drinking water. The rationale for using the same L-NAME concentration for NG and DM rats, even though the latter drank more water, was that in DM rats the production of renal *NO* was often found to be elevated [40]. Similar effectiveness of the higher L-NAME dose in DM animals was confirmed by measuring the response of the tissue *NO* signal to a post-experiment i.v. injection of L-NAME bolus (see the details below). In our previous studies, an analogous approach resulted in effective inhibition of endogenous *NO* production in rats on standard and high sodium intake; the latter showed higher water intake but also produced more *NO* [30].

### 4.3. Acute Experiment

#### 4.3.1. Surgical Preparations

Rats were anaesthetized with intraperitoneal sodium thiopental (Thipen, Samarth, India.), 100 mg/kg, which provided stable anaesthesia for at least 4 h. They were placed on a heated surgery table to maintain rectal temperature at about 37 °C. A polyethylene tube was placed in the trachea to ensure free airways. The jugular vein was cannulated for fluid infusions, and the carotid artery for mean arterial blood pressure (MABP) measurement (Stoelting blood pressure meter and transducers, Wood Dale, IL, USA). During surgery, 3% bovine serum albumin solution was infused i.v. at 3 mL/h to preserve plasma volume. The left kidney was exposed from a subcostal flank incision and placed in a plastic holder, similar to that used for micropuncture. The ureter was cannulated for timed urine collection and urine volume was determined gravimetrically. The details of the measurement of whole renal blood flow (RBF) (non-cannulating renal artery Transonic probe) as well as superficial cortical (CBF), outer medullary (OM-BF) and inner medullary (IM-BF) flows were as described previously [18,30].

For measurement of the tissue *NO* signal in the kidney, a needle-shaped ISO-NOP 200 sensor (0.2 mm in diameter), connected with Free Radical Analyser (TBR 4100, World Precision Instruments, Inc., Sarasota, FL, USA), was inserted vertically into the medulla, to the depth of 5–7 mm from the kidney surface. The details of *NO* measurement and calibration of the results were as described previously [18,29].

#### 4.3.2. Experimental Protocols

At the end of surgical preparation and after placement of the intrarenal probes and recovery from surgery, four 15 min urine collections (control periods) were made to determine baseline water, sodium, and total solute excretion rates in each of eight groups. After stabilization of renal haemodynamics, Theo (0.2 mmol/kg/h) or saline (S) was infused for 45 min, followed by recovery periods. This basic protocol was applied in the following experimental groups, (n = 7–8 per group):

1. Diabetic rats (0.9%NaCl i.v.), DM (0.9%NaCl);
2. Diabetic rats (Theo i.v.), DM (Theo);
3. Normoglycaemic rats (0.9%NaCl i.v.), NG (0.9%NaCl);
4. Normoglycaemic rats (Theo i.v.), NG (Theo);
5. Diabetic rats, L-NAME pre-treated (0.9%NaCl i.v.), DM+L-NAME (0.9%NaCl);
6. Diabetic rats, L-NAME pre-treated (Theo i.v.), DM+L-NAME (Theo);
7. Normoglycaemic rats, L-NAME pre-treated (0.9%NaCl i.v.), NG+L-NAME (0.9%NaCl);
8. Normoglycaemic rats, L-NAME pre-treated (Theo i.v.), NG+L-NAME (Theo).

### 4.4. Analytical Procedures and Calculations

BG was measured with a glucometer: ACCU-CHECK Active, Model GC (Roche, Mannheim, Germany). Urine volumes were determined gravimetrically. Urinary osmolality (U_osm_) was measured with the cryoscopic osmometer Osmomat 030 (Gonotec, Berlin, Germany). Urine sodium (U_Na_) and potassium (U_K_) concentrations were measured by a flame photometer (Flame Photometers, BWB Technologies, UK). Urine flow (V), the excretion of total solutes (U_osm_V) sodium (U_Na_V), and potassium (U_K_V) were calculated from the usual formulas and standardized to g kidney weight (U_X_V/g KW).

### 4.5. Statistics

Values are expressed as mean ± SEM. Data were analysed by repeated measurement ANOVA with Bonferroni’s test in case of multiple comparisons. *p* < 0.05 was considered as a significance level. When two sets of data within one group or two groups were compared, two-tailed Student’s *t*-test for paired or unpaired samples, respectively, was applied. With more than two data sets or groups, the significance of changes was evaluated by multivariate analysis of variance (ANOVA) with repeated measurements, followed by Tukey post hoc test (STATISTICA, version 10.0, StatSoft Inc., Tulsa, OK, USA).

## 5. Conclusions

The present study in female rats, using blockade of Ado and *NO*, alone or combined, provided some new insights regarding the interrelation of the two systems in normoglycaemic (NG) and diabetic (DM) animals.

1. In both male and female rats with intact *NO* synthesis, no tonic influence of the Ado system on the resistance of the peripheral vasculature was seen. However, in the kidneys of female rats, unlike in males, the vasoactive influence of the Ado system was not altered by hyperglycaemia.
2. In female rats, blockade of *NO* synthesis caused a slightly greater increase in MABP and a decrease in renal haemodynamics in NG compared with DM animals, indicating enhanced vasodilator influence of *NO* in diabetic females, but this was not found in an earlier study with diabetic males.
3. In *NO*-deficient NG and DM female rats, Ado receptor blockade induced comparable renal vasodilatation, suggesting a comparable vasoconstrictor influence of the Ado system in this sex, independent of the glycaemia level. However, the novel finding in NG female rats was an associated decrease in arterial pressure, of unclear origin.
4. Another novel and unexpected finding was that in female rats, both with intact or deficient *NO* synthesis, Ado receptor blockade had no or only a very slight impact on kidney tissue *NO,* in contrast to a distinct increase reported in males. Thus, in females only, Theo might somehow weaken rather than stimulate *NO* synthesis. The mechanism might be, in both sexes, the abolishment of the *NO*-inhibitory action of P1 receptors, presumably A1.
5. Lowered baseline renal excretion in female DM suggested stimulation of renal tubular fluid reabsorption, possibly due to the prevalence of antinatriuretic A1 over natriuretic A2 receptors. Remarkably, an opposite balance pattern between individual P1 receptor types emerged from the studies with males.

## Figures and Tables

**Table 1:** Body weight, data for blood, and plasma samples (upper section) and the data for 24 h observations in metabolic cages (lower section) for the samples obtained before (day 0) and 10 or 14 days after streptozotocin (STZ) or solvent injections in female Tac:Cmd:SD rats, normoglycaemic (NG) or diabetic (DM) before (day 10), and after L-NAME treatment for 4 days (box covered in grey depicts effects shown on the day 14).

		Days after Buffer or STZ Injection		
Parameter		0	10	14
Body weight	NG	239 ± 4	251 ± 4 *	258 ± 3 *
(g)	DM	237 ± 3	233 ± 5 #	233 ± 8 #
Glycaemia	NG	175 ± 2	171 ± 8	165 ± 13
(mg/dL)	DM	180 ± 3	493 ± 24 *#	526 ± 25 *#
Haematocrit	NG	44 ± 0	46 ± 1 *	46 ± 1 *
(%)	DM	44 ± 0	44 ± 1	45 ± 1 *
Plasma osmolality	NG	299 ± 6	299 ± 9	319 ± 4 *$
(mosmol/kg H_2_O)	DM	300 ± 3	311 ± 2 *	324 ± 5 *$
Plasma sodium concentration	NG	135 ± 1	131 ± 2	130 ± 2
(mmol/L)	DM	132 ± 2	127 ± 1 *#	126 ± 2 *
Plasma potassium concentration	NG	5.4 ± 0.4	5.8 ± 0.5	5.4 ± 0.7
(mmol/L)	DM	4.9 ± 0.3	5.2 ± 0.4	5.9 ± 0.5
Water intake	NG	31±1	30 ± 3	50 ± 26
(ml/24 h)	DM	25±2 #	95 ± 11 *#	126 ± 14 *#
Urine flow	NG	13±1	14 ± 2	15 ± 2
(ml/24 h)	DM	11±1	82 ± 10 *#	104 ± 10 *#
Urine osmolality	NG	1700±110	1550 ± 160	1400 ± 85
(mosmol/kg H_2_O)	DM	1730±250	1095 ± 55 *#	1095 ± 50 *
Total solute excretion	NG	27 ± 1	20 ± 1 *	25 ± 2
(mosmol/24 h)	DM	22 ± 1	113 ± 6 *#	126 ± 10 *#
Urine sodium excretion	NG	2.0 ± 0.1	1.4 ± 0.2 *	1.9±0.2
(mmol/24 h)	DM	1.6 ± 0.2	3.0 ± 0.3 *#	3.2±0.4 *#
Urine potassium excretion	NG	7.2 ± 0.5	5.2 ± 0.3 *	6.1 ± 0.6
(mmol/24 h)	DM	5.6 ± 0.8	4.3 ± 1.5	4.1 ± 1.9

L-NAME (NG-nitro-L-arginine methyl ester)—non-selective nitric oxide synthase inhibitor. *—significantly different from day 0, #—significantly different from NG, $—significantly different from day 10.

**Table 2:** Mean arterial blood pressure (MABP), heart rate (HR), renal haemodynamics and excretion in normoglycaemic (NG) or hyperglycaemic (DM) female rats, untreated or pre-treated with L-NAME.

Parameter	Pretreatment	NG	DM
MAP	Untreated	123 ± 3	124 ± 2
(mmHg)	L-NAME	149 ± 2 *	143 ± 4 *
HR	Untreated	349 ± 9	309 ± 6 †
(beat/min)	L-NAME	332 ± 6	283 ± 8 *†
RBF	Untreated	4.7 ± 0.3	3.8 ± 0.2 †
(ml/min/g of kidney weight)	L-NAME	2.7 ± 0.2 *	2.4 ± 0.1 *
RVR	Untreated	31 ± 3	34 ± 2
(mmHg min/ml)	L-NAME	69 ± 8 *	65 ± 2 *
CBF	Untreated	655 ± 25	535 ± 15†
(perfusion units)	L-NAME	480 ± 50 *	422 ± 9 *
V	Untreated	10.8 ± 1.4	7.0 ± 0.8 †
(µl/min/g of kidney weight)	L-NAME	7.3 ± 0.8 *	8.5 ± 3.2
U_osm_V	Untreated	7.3 ± 1.3	7.8 ± 1.3
(µosmol/min/g of kidney weight)	L-NAME	5.3 ± 0.6	9.6 ± 1.2 †
U_Na_V	Untreated	1.4 ± 0.4	0.5 ± 0.0 †
(µmol/min/g of kidney weight)	L-NAME	0.7 ± 0.1*	1.7 ± 0.3 *†
U_K_V	Untreated	1.0 ± 0.1	0.3 ± 0.0 †
(µmol/min/g of kidney weight)	L-NAME	0.6 ± 0.1 *	0.6 ± 0.1 *

RBF—whole-kidney blood flow, RVR—renal vascular resistance, CBF cortical blood flow (laser-Doppler flux), V—urine flow, U_osm_V, U_Na_V, U_K_V—the excretion of total solutes, sodium, and potassium, respectively. The values are means ± SEM; n = 7–8 for each group; *—significantly different from the corresponding untreated group; †—significantly different from the NG group.

**Table 3:** A comparison of baseline differences in mean arterial blood pressure (MABP), and parameters of renal excretion and urine concentration between female and male rats, normoglycaemic (NG) or with streptozotocin diabetes (DM), untreated (*NO-intact*) or pre-treated with L-NAME (*NO-deficient*).

		DM vs. NG	
	*NO Status*	Females	Males
MABP	*NO-intact*	↔	↔
	*NO-deficient*	(−) *	↔
V	*NO-intact*	(−) *	(+) *
	*NO-deficient*	(−) *	↔
U_osm_V	*NO-intact*	↔	(+) *
	*NO-deficient*	(+) *	(+) *
U_Na_V	*NO-intact*	(−) *	(+) *
	*NO-deficient*	(+) *	↔
U_K_V	*NO-intact*	(−) *	(−) *
	*NO-deficient*	↔	No data
U_osm_	*NO-intact*	(+) *	↔
	*NO-deficient*	(+) *	↔

V, urine excretion; UosmV, total solute excretion; U_Na_V and U_K_V, urine excretion of sodium and potassium; Uosm, urine osmolality. “(+)”/“(−)”, higher or lower mean value in DM vs. NG rats in the respective sex; ↔, no difference between DM and NG baseline in respective sex; *—significantly different vs. NG.

## Data Availability

Data is contained within the article and supplementary material.

## Supplementary Materials

The following supporting information can be downloaded at: https://www.mdpi.com/article/10.3390/ijms25147699/s1.
