# Supplementary material for: Sex Dependence in Control of Renal Haemodynamics and Excretion in Streptozotocin Diabetic Rats—Role of Adenosine System and Nitric Oxide
Source: Int J Mol Sci. 2024 Jul 13;25(14):7699. doi: 10.3390/ijms25147699 (PMC11276843; doi:10.3390/ijms25147699)
Supplement: Supplementary file 1 [file ijms-25-07699-s001.zip › Suppl data on Renal excretion ijms-3061539 _Kuczeriszka Dobrowolski .pdf]

Effects of theophylline on:

Urine flow (µl/min/per kidney weight) NG - normoglycaemic rats DM - diabetic rats

Exp. Periods	baseline				theophylline i.v.			recovery		
time [min]	-60	-45	-30	-15	15	30	45	60	75	90
NG-14	5,8	6,0	6,7	6,5	18,0	24,0	13,6	10,8	6,5	7,7
	8,6	12,1	8,7	11,8	30,3	37,6	22,5	12,8	9,8	11,5
	17,9	15,3	14,7	17,7	33,1	30,2	24,2	22,0	21,9	22,9
	9,8	15,9	28,7	48,2	60,3	44,8	23,2	14,1	11,3	15,3
	2,1	3,7	7,0	8,2	14,3	23,6	22,6	15,9	12,9	9,7
	2,7	2,9	4,9	6,3	2,5	8,5	21,7	6,4	3,8	10,7
	12,4	5,1	6,2	5,6	9,0	11,4	10,3	7,5	5,1	5,6
	8,3	11,8	19,7	24,2	50,3	63,9	50,4	36,5	23,0	26,2
NG-14 + L-NAME	4,7	6,6	7,8	10,7	15,3	24,1	12,6	5,6	13,5	13,1
	2,5	6,2	9,3	10,9	25,1	48,2	31,0	13,6	12,9	10,7
	3,9	6,6	8,4	7,5	14,9	27,4	25,3	18,7	12,7	8,7
	1,7	6,2	13,1	19,2	74,7	71,6	60,1	47,7	27,9	25,5
	7,6	12,2	13,0	13,3	21,7	30,0	22,9	20,5	15,5	15,1
	x	6,2	10,5	12,7	33,1	49,7	54,6	30,5	32,2	20,3
	1,7	1,2	3,2	5,0	12,1	24,8	26,4	23,1	17,3	18,2
	3,9	1,4	3,6	4,8	4,6	15,1	13,1	5,8	12,2	17,4
DM	2,7	3,8	6,9	8,1	10,3	10,5	9,8	8,8	9,2	10,3
	11,8	4,8	21,5	23,6	39,0	54,6	39,7	31,5	27,9	29,6
	4,6	4,8	5,4	8,4	12,8	18,5	15,2	9,1	10,5	8,3
	7,6	7,5	6,7	5,3	3,1	35,6	22,9	18,7	14,4	15,3
	8,2	4,5	4,7	3,5	4,5	8,2	9,2	14,0	15,3	15,6
	8,0	8,4	8,5	10,1	13,6	16,1	17,2	15,6	13,8	11,7
	5,0	4,3	5,0	4,9	4,8	6,9	7,0	9,8	10,9	9,8
	2,9	3,6	3,4	5,1	8,8	6,3	9,7	9,1	8,9	9,6
DM+L-NAME	3,7	6,9	9,1	10,1	13,2	17,3	12,5	11,2	10,1	8,7
	3,4	4,8	9,0	10,7	18,5	14,7	12,1	11,8	10,3	9,7
	1,4	0,6	1,4	2,6	4,6	3,5	5,6	2,5	0,4	1,4
	16,0	20,8	25,6	21,6	21,8	26,4	15,0	10,9	7,2	7,6
	6,4	5,8	5,5	7,1	10,7	15,6	6,8	1,6	x	x
	8,1	6,7	9,1	7,7	10,9	17,1	17,6	14,1	15,2	11,1

x - recording failed

Effects of theophylline on:

Urine osmolality (mosmol/kg H_2O) NG - normoglycaemic rats DM - diabetic rats

Exp. Periods	baseline				theophylline i.v.			recovery		
time [min]	-60	-45	-30	-15	15	30	45	60	75	90
NG-14	673	724	771	848	701	725	780	885	890	895
	713	655	673	676	577	615	756	925	1040	970
	478	541	545	530	506	556	586	597	594	587
	725	575	490	480	435	520	660	800	835	735
	1135	985	835	825	830	690	790	895	925	970
	800	1086	1131	1153	1155	938	595	879	1068	792
	590	635	640	685	725	795	840	860	880	905
	760	665	545	480	385	385	450	530	620	585
NG-14 + L-NAME	611	499	491	529	571	607	785	1103	1016	829
	554	577	632	598	455	396	452	618	686	731
	690	700	710	810	850	735	770	860	945	995
	620	574	576	521	462	436	514	544	621	655
	790	465	515	520	500	710	715	695	675	670
	x	400	450	445	390	375	390	465	540	565
	455	845	435	405	350	335	340	375	410	430
	595	505	688	765	836	700	788	1014	835	825
DM	1068	964	999	1090	1150	1292	1362	1295	1224	1130
	1135	712	797	886	823	839	885	867	885	884
	1380	1081	1093	989	980	950	1018	1212	1140	1107
	1569	1360	982	947	958	932	728	780	850	832
	669	721	739	780	860	1043	1098	817	747	907
	1077	1063	1090	1149	1125	1126	1092	1081	1155	1169
	985	982	978	1049	987	1087	1152	1114	1149	1077
	935	895	875	1000	1048	1198	1095	1180	1235	1210
DM+L-NAME	1040	1010	965	955	1050	1170	1500	1710	1420	1250
	820	740	790	795	755	835	945	970	1005	995
	1257	x	x	1321	1271	1506	1662	1755	x	1445
	784	784	693	693	735	738	906	1008	1035	1016
	1832	1707	1622	1522	1265	1151	877	730	x	x
	865	936	915	921	908	834	884	939	969	1003

x - recording failed

Effects of theophylline on:

Total solute excretion (µosmol/min/ per kidney weight) NG - normoglycaemic rats DM - diabetic rats

Exp. Periods	baseline				theophylline i.v.			recovery		
time [min]	-60	-45	-30	-15	15	30	45	60	75	90
NG-14	3,9	4,3	5,2	5,5	12,6	17,4	10,6	9,6	5,8	6,9
	6,1	7,9	5,8	8,0	17,5	23,1	17,0	11,9	10,2	11,2
	8,6	8,3	8,0	9,4	16,8	16,8	14,2	13,1	13,0	13,5
	7,1	9,1	14,0	23,1	26,2	23,3	15,3	11,3	9,4	11,3
	2,4	3,6	5,8	6,8	11,8	16,3	17,8	14,2	11,9	9,4
	2,2	3,2	5,5	7,3	2,9	8,0	12,9	5,6	4,0	8,4
	7,3	3,2	4,0	3,8	6,5	9,1	8,7	6,5	4,5	5,1
	6,3	7,8	10,7	11,6	19,4	24,6	22,7	19,3	14,3	15,3
NG-14 + L-NAME$_4$	2,9	3,3	3,8	5,6	8,7	14,7	9,9	6,2	13,8	10,9
	1,4	3,6	5,9	6,5	11,4	19,1	14,0	8,4	8,9	7,8
	2,7	4,6	5,9	6,1	12,6	20,2	19,5	16,1	12,0	8,7
	1,1	3,6	7,5	10,0	34,5	31,2	30,9	26,0	17,3	16,7
	6,0	5,7	6,7	6,9	10,9	21,3	16,4	14,2	10,5	10,1
	X	2,5	4,7	5,6	12,9	18,7	21,3	14,2	17,4	11,5
	0,8	1,0	1,4	2,0	4,3	8,3	9,0	8,7	7,1	7,8
	2,3	0,7	2,5	3,6	3,8	10,6	10,3	5,9	10,2	14,4
DM	2,8	3,6	6,9	8,9	11,8	13,5	13,4	11,4	11,2	11,6
	13,4	3,4	17,1	20,9	32,1	45,8	35,1	27,3	24,7	26,1
	6,3	5,2	6,0	8,3	12,5	17,6	15,4	11,0	12,0	9,2
	11,9	10,2	6,6	5,0	3,0	33,2	16,7	14,6	12,2	12,8
	5,5	3,2	3,4	2,7	3,9	8,5	10,1	11,5	11,4	14,1
	8,7	8,9	9,3	11,6	15,3	18,1	18,7	16,9	15,9	13,7
	4,9	4,3	4,9	5,2	4,8	7,5	8,0	10,9	12,6	10,5
	2,7	3,2	3,0	5,1	9,2	7,5	10,6	10,8	11,0	11,6
DM+L-NAME$_4$	3,9	7,0	8,8	9,6	13,8	20,3	18,7	19,2	14,3	10,9
	2,8	3,6	7,1	8,5	14,0	12,3	11,4	11,4	10,3	9,7
	1,8	x	x	3,4	5,9	5,2	9,3	4,5	x	2,0
	12,5	16,3	17,7	15,0	16,0	19,5	13,6	11,0	7,5	7,8
	11,8	9,9	8,9	10,8	13,5	17,9	6,0	1,2	x	x
	7,0	6,3	8,3	7,1	9,9	14,3	15,5	13,3	14,7	11,2

x - recording failed

Effects of theophylline on:

Sodium Excretion (µmol/min/per kidney weight) NG - normoglycaemic rats DM - diabetic rats

Exp. Periods	baseline				theophylline i.v.			recovery		
time [min]	-60	-45	-30	-15	15	30	45	60	75	90
NG-14	0,5	0,5	0,6	0,6	2,6	4,5	3,0	2,5	1,5	1,8
	0,5	1,0	0,7	0,9	4,2	6,7	4,5	3,1	2,3	2,7
	2,3	1,8	1,7	2,2	5,0	5,1	4,3	3,8	4,2	4,2
	1,1	1,8	3,6	6,0	8,5	7,2	4,6	3,0	2,5	2,8
	0,2	0,2	0,5	1,2	2,0	5,2	5,9	2,9	2,7	2,2
	0,2	0,3	0,5	0,7	0,3	0,9	2,4	1,0	0,7	1,6
	1,1	0,4	0,4	0,4	0,9	1,7	1,7	1,2	0,7	0,7
	0,7	0,9	2,1	2,8	6,1	8,1	7,9	6,3	4,3	4,6
NG-14 + L-NAME$_4$	0,1	0,1	0,1	0,2	0,7	2,2	1,5	0,7	2,0	2,2
	0,0	0,2	0,5	0,8	2,0	5,2	3,7	2,0	1,9	1,7
	0,4	0,7	0,8	0,8	2,1	5,5	5,8	4,7	2,9	2,4
	0,0	0,2	0,7	1,5	10,2	10,7	10,1	7,9	5,0	4,5
	0,7	0,8	1,1	1,1	2,3	4,0	3,9	3,3	2,5	2,3
	x	0,5	0,9	1,2	3,9	8,1	8,3	5,2	6,1	3,8
	0,1	0,1	0,2	0,2	0,7	3,0	4,2	4,2	2,7	3,1
	0,2	x	0,2	0,3	0,4	1,5	1,7	1,0	1,9	2,6
DM	0,1	0,1	0,1	0,2	0,5	0,7	0,9	0,7	0,8	1,0
	0,3	0,2	0,5	0,6	1,4	4,0	2,6	1,6	1,3	1,8
	0,3	0,3	0,3	0,6	1,5	2,6	2,4	1,3	1,5	1,0
	0,5	0,5	0,5	0,4	0,3	4,7	3,7	2,9	2,3	2,3
	0,6	0,4	0,4	0,2	0,3	0,5	0,6	1,2	1,5	1,3
	0,4	0,6	0,6	0,6	0,9	1,2	1,5	1,3	1,2	0,9
	0,4	0,5	0,7	0,7	0,5	0,7	0,9	0,6	0,5	0,6
	0,2	0,3	0,3	0,7	1,6	0,9	1,6	1,3	1,3	1,4
DM+L-NAME$_4$	0,7	1,7	2,2	2,3	2,7	3,5	2,9	2,6	2,0	1,5
	0,2	0,4	1,0	1,4	2,7	2,3	1,9	1,8	1,8	2,0
	0,1			0,3	0,6	0,6	??	0,4		0,2
	2,1	2,7	4,7	4,0	4,0	4,9	2,7	1,8	1,0	1,0
	0,9	0,5	0,7	0,5	1,1	0,6	0,1	0,0	x	x
	1,6	1,4	2,0	1,7	2,4	3,6	3,1	2,0	2,2	1,4

x - recording failed

Effects of theophylline on:

Potassium Excretion (µmol/min/per kidney weight)   NG - normoglycaemic rats   DM - diabetic rats

Exp. Periods	baseline				theophylline i.v.			recovery		
time [min]	-60	-45	-30	-15	15	30	45	60	75	90
NG-14	0,5	0,7	0,9	1,0	1,6	1,8	1,3	1,0	0,6	0,7
	0,7	1,4	1,1	1,4	2,5	2,9	2,0	1,2	1,1	1,2
	1,3	1,4	1,3	1,5	2,3	2,2	1,7	1,4	1,3	1,4
	1,0	1,1	1,5	1,7	1,6	1,3	1,2	1,2	1,2	1,1
	0,6	0,8	1,1	1,3	2,3	2,8	3,4	1,9	1,7	1,2
	0,1	0,3	0,6	0,8	0,3	1,0	1,5	0,8	0,5	0,8
	1,1	0,4	0,5	0,5	1,0	1,4	1,2	0,9	0,5	0,5
	1,1	1,2	1,7	1,7	2,3	2,2	2,1	1,8	1,4	1,4
NG-14 + L-NAME	0,2	0,3	0,5	0,8	1,8	2,9	1,7	0,9	2,0	1,4
	0,0	0,2	0,5	0,8	1,3	2,3	1,6	0,9	1,0	0,8
	0,3	0,8	1,0	1,1	2,0	2,6	2,2	1,8	1,3	0,9
	0,0	0,5	1,0	1,3	2,6	2,1	2,0	1,8	1,3	1,3
	0,8	0,9	1,0	1,0	1,5	2,1	1,7	1,4	1,1	1,0
	x	0,5	0,8	0,6	1,5	1,7	2,4	1,7	1,9	1,0
	0,1	x	0,2	0,3	0,8	1,0	0,9	0,9	0,7	0,9
	0,2	0,1	0,3	0,4	0,5	1,7	1,5	0,7	1,1	1,6
DM	0,1	0,2	0,4	0,6	1,1	1,2	1,2	1,0	1,1	1,2
	0,4	0,1	0,4	0,5	0,9	1,4	1,0	0,8	0,6	0,7
	0,2	0,2	0,3	0,7	1,2	1,6	1,4	0,8	1,0	0,7
	0,5	0,4	0,5	0,4	0,3	2,2	1,6	1,3	1,1	1,0
	0,3	0,2	0,2	0,2	0,3	0,8	0,9	0,9	0,9	1,0
	0,3	0,6	0,7	0,8	1,1	1,2	1,2	1,0	0,8	0,6
	0,1	0,1	0,1	0,1	0,1	0,2	0,3	0,3	0,2	0,3
	0,1	0,2	0,2	0,5	1,0	0,7	1,0	0,9	0,9	0,9
DM+L-NAME	0,2	0,4	0,6	0,7	0,8	1,2	1,1	1,1	0,9	0,6
	0,2	0,4	0,9	1,1	1,9	1,5	1,3	1,3	1,3	1,3
	0,1	x	x	0,5	0,9	0,7	x	0,4	x	0,2
	0,9	1,1	1,5	1,3	1,0	0,8	0,8	0,6	0,2	0,2
	0,4	0,3	0,4	0,6	0,7	0,2	0,1	0,1	x	x
	0,5	0,5	0,6	0,6	0,8	1,2	1,1	0,8	0,8	0,6

x - recording failed

Left kidney weight (g)

NG - normoglycaemic rats

DM - diabetic rats

NG-14	1,270
	1,059
	1,240
	1,091
	0,925
	1,117
	1,153
	1,009
NG-14 + L-NAME	1,525
	1,192
	1,139
	1,154
	1,044
	0,993
	1,309
	1,112
DM	1,317
	1,145
	1,406
	1,287
	1,464
	1,392
	1,288
	1,474
DM+L-NAME	1,281
	1,284
	1,57
	1,402
	1,293
	1,31
	1,331
