# Supplementary material for: Sex Dependence in Control of Renal Haemodynamics and Excretion in Streptozotocin Diabetic Rats—Role of Adenosine System and Nitric Oxide
Source: Int J Mol Sci. 2024 Jul 13;25(14):7699. doi: 10.3390/ijms25147699 (PMC11276843; doi:10.3390/ijms25147699)
Supplement: Supplementary file 1 [file ijms-25-07699-s001.zip › Suppl data on Renal haemodynamics ijms-3061539 _Kuczeriszka Dobrowolski.pdf]

Effects of theophylline on blood pressure in anaesthetized Sprague-Dawley rats.

Mean Blood Pressure (mmHg)

Exp. Periods	baseline				theophylline i.v.			recovery		
Exp. Group/time [min]	-60	-45	-30	-15	15	30	45	60	75	90
NG-14	128	130	128	129	126	121	112	114	121	116
	133	135	133	133	133	133	133	136	133	126
	88	86	94	97	97	97	95	100	100	101
	131	131	130	130	128	128	127	128	129	130
	127	126	126	127	127	126	126	124	118	122
	133	133	132	133	132	134	138	137	134	132
	94	91	90	93	94	97	95	93	89	91
	149	145	145	145	143	142	143	144	144	144
NG-14 + L-NAME	153	156	158	162	158	155	149	140	118	120
	156	154	151	150	148	148	148	146	146	146
	135	135	135	135	135	133	135	137	139	139
	163	159	155	151	144	140	138	140	140	139
	152	150	149	147	141	138	140	140	140	139
	150	151	151	152	151	150	152	147	147	148
	146	142	139	136	130	133	135	140	143	140
DM	142	142	140	137	137	134	136	136	136	136
	121	122	119	122	121	119	113	108	112	113
	131	129	130	128	125	123	122	121	119	119
	108	110	115	117	119	120	118	118	118	118
	124	123	119	115	116	119	119	123	124	122
	122	122	118	114	112	116	115	116	119	122
	121	120	119	117	116	117	114	114	115	116
	130	130	130	130	128	127	130	131	130	130
DM+L-NAME	143	144	145	152	155	153	151	152	153	153
	146	146	146	146	146	146	143	141	139	139
	118	116	115	114	113	112	112	112	111	110
	148	144	143	143	142	142	141	142	141	140
	166	164	164	164	161	157	151	149	148	149

Effects of theophylline on blood pressure in anaesthetized Sprague-Dawley rats.

Heart rate (beats per min)

Exp. Periods	baseline				theophylline i.v.			recovery		
Exp. Group/time [min]	-60	-45	-30	-15	15	30	45	60	75	90
NG-14	403	402	397	395	417	475	476	460	463	468
	350	349	340	336	373	373	432	438	430	415
	422	423	427	422	469	476	471	470	456	457
	382	373	369	368	393	430	365	371	367	388
	365	367	376	385	408	426	446	433	423	452
	316	318	318	316	325	365	442	433	430	398
	279	282	278	280	294	318	329	327	327	328
	295	285	285	268	254	236	224	208	202	x
NG-14 + L-NAME	x	x	x	x	x	x	x	x	x	x
	337	339	343	344	359	410	441	454	453	448
	315	318	320	316	344	401	429	427	425	430
	374	376	368	366	367	396	415	418	377	x
	366	356	353	346	351	392	419	424	418	410
	317	320	317	318	321	342	311	330	330	317
	290	285	299	290	295	305	284	295	x	x
DM	329	296	269	281	295	362	360	344	340	338
	279	290	276	274	321	333	333	323	313	318
	291	282	284	286	315	334	339	339	319	315
	309	336	336	330	325	340	338	350	361	354
	397	389	368	340	360	410	433	450	465	472
	262	303	301	298	309	331	340	343	337	331
	290	294	300	296	307	333	344	358	346	347
	332	329	326	325	342	366	382	379	373	372
DM+L-NAME	268	281	279	280	292	331	358	371	361	358
	344	339	337	340	349	381	410	416	401	400
	298	295	295	292	306	323	344	339	326	333
	272	265	262	266	276	295	351	355	340	349
	239	239	239	239	254	290	332	352	338	334

x - recording failed

Effects of theophylline on blood pressure in anaesthetized Sprague-Dawley rats.

Renal Blood Flow (ml/min/per kidney weight)

Exp. Periods	baseline				theophylline i.v.			recovery		
Exp. Group/time [min]	-60	-45	-30	-15	15	30	45	60	75	90
NG-14	4,2	4,4	4,6	4,7	5,4	5,9	5,7	5,5	5,5	5,4
	2,1	2,0	2,0	1,9	2,1	2,1	2,4	2,2	2,2	2,2
	7,4	8,3	7,6	6,8	7,4	7,8	**7,8**	7,5	7,1	7,0
	5,0	5,3	5,5	5,5	5,8	5,5	5,3	5,3	5,2	5,1
	6,9	6,7	6,4	5,9	5,8	5,9	5,9	5,8	5,5	4,8
	3,2	3,4	3,4	3,5	3,5	3,4	3,8	4,1	4,2	4,8
	4,7	4,3	4,2	4,0	4,5	5,1	**5,1**	4,9	4,9	4,9
	4,3	4,3	4,3	4,3	4,4	4,8	**5,1**	5,2	5,2	5,3
NG-14 + L-NAME	2,9	2,9	2,9	2,9	3,2	3,6	**3,8**	4,0	4,2	**4,3**
	4,2	4,5	4,6	4,6	4,5	4,8	**5,0**	5,0	5,0	4,9
	x	x	x	x	x	x	x	x	x	x
	3,0	3,0	3,0	3,0	3,1	3,2	**3,2**	**3,2**	3,0	3,0
	3,1	3,0	3,1	3,1	3,4	3,6	**3,6**	**3,6**	3,5	3,6
	1,0	1,0	1,0	1,0	1,0	1,1	1,1	1,1	1,1	1,1
	1,9	2,1	2,2	2,3	2,5	2,6	2,6	**2,8**	2,5	2,3
DM	5,4	5,4	5,6	5,5	6,3	6,5	6,3	6,2	5,9	5,8
	4,6	4,7	4,6	4,8	5,0	5,1	4,8	4,2	4,1	4,0
	3,9	4,0	4,0	4,0	4,8	5,1	5,0	4,9	4,7	4,6
	3,8	3,9	4,0	4,0	4,3	4,7	4,9	4,7	4,5	4,4
	3,0	3,1	3,4	3,5	3,5	3,3	3,0	2,7	2,6	2,5
	2,8	2,7	2,9	3,0	3,6	3,9	4,4	5,0	4,9	5,0
	2,3	2,3	2,4	2,2	2,2	2,3	2,4	2,45	2,44	2,5
	4,1	4,2	4,3	4,1	4,1	4,4	4,5	4,5	4,7	4,7
DM+L-NAME	2,9	3,0	3,1	3,1	2,5	2,7	2,9	3,0	2,9	2,9
	2,7	2,7	2,7	2,6	2,8	3,0	**3,2**	3,2	3,1	3,0
	2,4	2,5	2,6	2,7	3,0	3,1	3,0	2,7	2,6	2,6
	1,6	1,6	1,6	1,6	1,8	2,2	2,4	2,4	2,3	2,3
	2,1	2,1	2,0	1,9	2,0	2,6	2,9	2,9	2,7	2,7

x - recording failed

Effects of theophylline on blood pressure in anaesthetized Sprague-Dawley rats.

CBF laser-Doppler probe measurements

Cortical blood flow (Perfusion Units)

Exp. Group/time [min]	baseline				theophylline i.v.			recovery		
time [min]	-60	-45	-30	-15	15	30	45	60	75	90
NG-14	684	671	685	698	735	763	744	714	708	703
	318	323	331	333	356	356	386	367	356	362
	702	736	732	697	738	768	761	723	638	617
	594	605	616	622	642	615	603	604	599	596
	835	826	809	795	817	842	887	905	810	614
	781	791	774	796	656	587	672	848	832	849
	694	687	706	687	729	767	795	777	753	747
	612	618	620	637	683	699	730	737	741	734
NG-14 + L-NAME	530	538	538	538	521	562	589	679	657	549
	508	491	512	517	557	629	655	656	650	641
	496	546	572	584	625	619	616	594	575	581
	573	571	567	579	606	624	626	611	598	596
	551	545	556	550	577	596	599	590	580	579
	312	302	306	327	362	379	371	354	338	333
	218	227	243	258	276	318	349	330	341	349
DM	556	559	549	533	525	524	519	519	517	520
	x	x	x	x	x	x	x	x	x	x
	511	518	515	515	594	632	627	606	588	579
	671	653	628	653	682	734	755	750	729	707
	x	x	x	x	x	x	x	x	x	x
	x	x	x	x	x	x	x	x	x	x
	491	480	441	351	307	334	320	287	304	341
	577	575	571	576	566	589	603	613	615	613
DM+L-NAME	485	479	489	505	528	571	608	615	608	609
	436	456	451	470	470	500	523	537	528	512
	417	426	420	424	462	446	435	399	376	373
	395	398	399	397	426	469	493	481	466	456
	388	350	334	331	327	427	447	458	449	444
	x	x	x	x	x	x	x	x	x	x
	442	413	415	403	411	433	469	462	434	412

x - recording failed

Effects of theophylline on blood pressure in anaesthetized Sprague-Dawley rats.

OMBF laser-Doppler probe measurements

Outer medullary blood flow (Perfusion Units)

Exp. Group/time [min]	baseline				theophylline i.v.			recovery		
time [min]	-60	-45	-30	-15	15	30	45	60	75	90
NG-14	130	122	120	120	127	136	128	119	106	89
	183	174	177	159	161	161	174	181	178	184
	347	365	389	393	376	388	380	382	376	382
	274	246	265	253	234	220	223	229	224	205
	191	180	174	169	169	171	174	181	188	175
	69	63	63	66	60	55	60	65	79	76
	x	220	213	208	221	313	351	349	306	300
	160	160	157	149	142	138	145	145	154	162
NG-14 + L-NAME	315	306	296	292	307	323	339	352	343	344
	152	157	163	162	143	148	182	185	161	155
	x	x	x	x	x	x	x	x	x	x
	179	188	201	184	171	134	129	130	131	143
	201	194	192	184	190	210	218	201	193	181
	236	241	234	225	251	246	263	268	247	243
	123	117	127	122	111	113	115	118	117	120
	79	73	66	65	62	63	60	67	62	59
DM	304	262	244	220	214	240	281	284	275	216
	x	164	137	140	156	177	175	173	170	159
	105	103	94	92	85	89	97	108	110	107
	155	172	170	178	203	220	221	215	208	201
	312	308	318	310	297	295	293	216	227	224
	261	256	229	233	253	245	244	261	239	231
	64	68	66	65	61	55	55	59	52	51
	111	106	104	100	95	98	98	96	95	94
DM+L-NAME	193	202	201	201	186	195	219	213	200	202
	75	69	67	69	70	78	93	102	109	119
	327	317	318	306	343	373	359	313	327	338
	111	108	109	110	119	128	143	141	139	138
	137	143	141	143	141	x	x	x	x	x
	180	180	190	189	184	200	231	236	222	218

x - recording failed

Effects of theophylline on blood pressure in anaesthetized Sprague-Dawley rats.

IMBF laser-Doppler probe measurements

Inner medullary blood flow (Perfusion Units)

Exp. Group/time [min]	baseline				theophylline i.v.			recovery		
time [min]	-60	-45	-30	-15	15	30	45	60	75	90
NG-14	252	256	259	280	257	256	276	290	296	282
	104	111	98	108	107	107	116	120	129	106
	153	170	158	163	173	186	193	199	204	203
	122	100	95	92	92	100	111	112	113	111
	x	x	x	x	x	x	x	x	x	x
	196	192	183	187	120	88	107	165	177	148
	357	347	360	364	371	387	410	400	400	370
	178	174	172	177	157	157	168	186	192	184
NG-14 + L-NAME	103	92	91	93	76	103	108	112	118	122
	117	117	117	117	117	117	110	118	110	101
	x	x	x	x	x	x	x	x	x	x
	245	226	216	218	219	221	209	221	224	226
	225	208	203	201	167	115	127	125	123	116
	161	147	143	137	169	163	125	119	107	103
	101	101	99	99	98	100	102	102	100	97
	193	198	202	202	209	217	211	220	210	201
DM	138	131	119	112	104	103	98	94	95	87
	x	250	235	230	244	250	265	269	266	272
	98	87	82	76	83	116	149	170	177	173
	160	196	152	139	120	129	114	103	98	94
	218	221	204	216	222	227	220	184	201	243
	218	209	210	214	222	227	238	238	225	222
	99	107	107	104	109	71	65	67	63	61
DM+L-NAME	120	112	117	115	126	110	**124**	131	126	126
	221	227	224	224	210	203	213	209	199	194
	198	197	219	225	180	180	179	207	184	181
	126	128	125	126	134	154	183	187	180	177
	94	98	103	102	101	x	x	x	x	x
	x	x	x	x	x	x	x	x	x	x
	191	188	207	198	194	227	270	297	271	262

x - recording failed

Effects of theophylline on blood pressure in anaesthetized Sprague-Dawley rats.
Tissue NO relative changes *vs.* period "-15" (%)

Exp. Periods	baseline				theophylline i.v.			recovery		
Exp. Group/time [min]	-60	-45	-30	-15	15	30	45	60	75	90
NG-14	-2	-7	-3	0	-4	-2	-4	-3	0	-3
	-4	-1	-13	0	-5	-5	-6	-13	-10	-9
	6	3	3	0	3	1	-4	-2	-6	-6
	6	5	3	0	-3	-2	2	10	7	2
	0	0	0	0	10	12	6	3	6	-5
	4	3	1	0	0	-2	-4	-7	-9	-10
	4	0	0	0	0	-3	-2	-3	0	4
	2	0	-1	0	-5	-8	-10	-16	-17	-16
NG-14 + L-NAME	3	1	1	0	0	-1	-1	0	0	-1
	10	8	3	0	0	-4	-6	-8	-10	-10
	x	x	x	x	x	x	x	x	x	x
	-1	-2	-2	0	1	6	8	10	6	6
	6	3	1	0	1	1	2	2	3	4
	8	1	0	0	-4	-5	-9	-10	-10	-10
	2	1	0	0	-2	-5	-7	-10	-10	-11
DM	0	0	1	0	3	5	4	4	3	2
	1	5	2	0	0	-2	-5	-7	-8	-10
	-2	0	0	0	3	4	4	5	3	3
	3	1	0	0	-1	-3	-3	-3	-3	-3
	8	6	3	0	0	1	0	1	-1	-3
	-6	-4	0	0	1	2	1	1	0	-3
	x	x	x	x	x	x	x	x	x	x
	-3	0	0	0	3	3	4	7	8	7
DM+L-NAME	9	4	2	0	-2	-4	-5	-6	-4	-6
	3	0	0	0	2	2	3	5	2	0
	3	1	-1	0	0	-1	0	-3	-7	-7
	0	1	1	0	3	x	x	x	x	x
	0	1	1	0	0	1	2	4	1	0

x - recording failed
